# Fabry Disease in Slovakia: How the Situation Has Changed over 20 Years of Treatment

**DOI:** 10.3390/jpm12060922

**Published:** 2022-06-01

**Authors:** Katarina Jurickova, Petra Jungova, Robert Petrovic, Slavomira Mattosova, Tereza Hlavata, Ludmila Kostalova, Anna Hlavata

**Affiliations:** 1 Department of Paediatrics, Faculty of Medicine Comenius University in Bratislava and National Institute of Children’s Diseases in Bratislava, 83340 Bratislava, Slovakia; ludka.kostalova@gmail.com (L.K.); anna.hlavata@nudch.eu (A.H.); 2 Centre for Inborn Errors of Metabolism, National Institute of Children’s Diseases in Bratislava, 83340 Bratislava, Slovakia; 3 Institute of Medical Biology, Genetics and Clinical Genetics, Faculty of Medicine Comenius University and University Hospital in Bratislava, 81107 Bratislava, Slovakia; petra.jungova@gmail.com (P.J.); robert.petrovic@fmed.uniba.sk (R.P.); smattosova@gmail.com (S.M.); 4 Adult Congenital Heart Disease Department, Cardiology and Angiology Clinic, Slovak Medical University and National Institute For Cardiovascular Diseases, 83348 Bratislava, Slovakia; tereza.hlavata@gmail.com

**Keywords:** Fabry disease, lysosomal storage disorder, multisystemic manifestation, diagnostics, genetic variants of *GLA*, therapy

## Abstract

Fabry disease (FD, OMIM#301500) is a rare inborn error of the lysosomal enzyme α-galactosidase (α-Gal A, EC 3.2.1.22) and results in progressive substrate accumulation in tissues with a wide range of clinical presentations. Despite the X-linked inheritance, heterozygous females may also be affected. Hemizygous males are usually affected more severely, with an earlier manifestation of the symptoms. Rising awareness among health care professionals and more accessible diagnostics have positioned FD among the most-common inherited metabolic diseases in adults. An early and correct diagnosis of FD is crucial with a focus on personalised therapy. Preventing irreversible destruction of vital organs is the main goal of modern medicine. The aim of this study was to offer a complex report mapping the situation surrounding FD patients in Slovakia. A total of 48 patients (21 males, 27 females) with FD are registered in the Centre for Inborn Errors of Metabolism in Bratislava, Slovakia. In our cohort, we have identified three novel pathogenic variants in five patients. Three patients presented with the frameshift mutation c.736delA, and two others presented with the missense mutations c.203T>C, c.157A>C. Moreover, we present a new clinical picture of the pathogenic variant c.801+1G>A, which was previously described and associated with the renal phenotype.

## 1. Introduction

Fabry disease (FD, OMIM#301500) is an X-linked lysosomal storage disorder (LSD), caused by the deficiency of α-galactosidase A (α-Gal A, EC 3.2.1.22), which leads to a progressive accumulation of the globotriaosylceramide (Gb3) and its derivatives (e.g., lyso-Gb3) in various tissues [1]. The disease has attracted a lot of interest over the last years, since enzyme replacement therapy (ERT), as a treatment, has become widely available as of 2001 [2].

In the past, inborn errors of metabolism were considered paediatric diseases. With rising awareness and rising numbers of diagnosed patients, mainly due to screening in high-risk populations, the situation has changed [3].

The study of Sirrs et al., published in 2015 concerning the frequencies of different inborn metabolic errors in adult metabolic centres, reported that FD is the second most-common diagnosis [4].

FD presents with highly variable symptomatology, ranging from patients who are asymptomatic to those severely affected with multiorgan damage. Classic FD in males is characterised by the onset of symptoms in childhood or adolescence, absent or severely reduced (<1% of normal) α-Gal A enzyme activity, and microvascular endothelial Gb3 accumulation. Typical manifestations include cutaneous lesions (angiokeratoma), hypohidrosis, peripheral neuropathy (with acral pain and painful febrile crises), corneal deposits (cornea verticillata), gastrointestinal disturbances (bloating, diarrhoea, abdominal pain), and albuminuria. At a later age, progressive kidney disease, hypertrophic cardiomyopathy, and cerebrovascular disease (including stroke) can occur. However, a larger group of patients has later-onset phenotypes with varying levels of residual α-Gal A activity, ages of onset, and manifestations. Patients with later-onset or non-classical FD may present with one single non-specific symptom, such as chronic kidney disease or left ventricular hypertrophy (LVH) [5,6].

Heterozygous females may also be affected, and they generally demonstrate a more variable and attenuated phenotype. The onset and the severity of symptoms in women primarily depends on the random pattern of inactivation of one of the two X chromosomes [7,8]. In both males and females, life expectancy is reduced, although this is more apparent in males [9,10].

This variability is challenging for physicians in determining prognosis, and consequently, a diagnosis of FD does not automatically merit the initiation of FD-specific treatment with enzyme replacement therapy (ERT) or chaperone therapy. Instead, physicians must monitor patients regularly to identify signs that may warrant treatment initiation. The decision whether to treat may be problematic and unclear [10].

Recently, enhanced deposits of globotriaosylsphingosine (LysoGb3) have been shown to be a characteristic feature of FD. The deacylated Gb3, LysoGb3, also known as globotriaosylsphingosine, has been reported as a potential diagnostic tool in both classic and uncertain cases. LysoGb3 serves as a useful biomarker to improve the diagnosis of FD heterozygotes and for therapeutic evaluation and monitoring [11,12].

α-Gal A activity testing alone is a sufficient diagnostic tool for male patients; however, confirmation of the disease-causing *GLA* mutation is important to help predict the disease phenotype and to rule out benign polymorphisms that also can cause reduced levels of α-Gal A activity, and it also permits targeted testing of at-risk family members. In female patients, the demonstration of the presence of a disease-causing mutation in the *GLA* gene is ultimately required. Due to random X-chromosomal inactivation, the plasma enzyme activity is often found within the normal range, although leukocyte α-Gal A activity may be lower, as in the reference group [9,13].

To date, over 1000 pathogenic variants have been identified in the *GLA* gene; however, no frequent mutations were described, and it is difficult to establish a genotype–phenotype correlation. Most of the pathogenic *GLA* mutations are private, occurring in a single or a few families. Moreover, intrafamilial and interfamilial variability in the age of the disease onset and progression exists. Many genetic variants of unknown significance (GVUS) have been identified during screening programs. GVUS is an additional challenge and further studies must be conducted [5,13,14].

Fabry disease affects all ethnicities, with some geographical clusters based on the founder mutations’ frequency. The reported prevalence of FD varies according to the screening method employed. Previously, data based on clinically diagnosed cases of predominantly classic FD suggested prevalence figures of 1 in 117,000 [15]. In contrast, neonatal screening programs have reported an unexpectedly high incidence rate, ranging from 1:1250 male live births [16] to around 1:9000 live births [17,18], depending on the geographical region, study, and definition of FD.

The prevalence of Fabry disease has been extensively studied in patients from various nations and ethnicities. Until now, there have been no published reports concerning the situation of Fabry disease in Slovakia.

## 2. Materials and Methods

In the Centre for Inborn Errors of Metabolism in the National Institute of Children’s Diseases in Bratislava, we performed a retrospective study over a period of 23 years (1999–2021). During the time of the study, 48 Slovak Fabry patients (21 males and 27 females) have been included. The median (range) age at the time of diagnosis for males was 50.0 (4.0–78.0) and for females was 32.5 (0.0–85). Baseline demographic and clinical characteristics were recorded, including gender, age at diagnosis, *GLA* variant, phenotype, and the type of screening in which the patient was identified. In the treated group, we recorded age at symptom onset, age at diagnosis, age at ERT initiation, duration of ERT, effect of the treatment, time from onset of the symptom to diagnosis, time from diagnosis to treatment, LysoGB3 values prior to and after ERT treatment (available since 2017), and survival. Clinical assessment focused on medical history, and FD-related signs and symptoms were obtained. Renal (proteinuria, albuminuria, serum creatinine, urine albumin-to-creatinine ratio, estimated glomerular filtration rate) and cardiac (N-terminal prohormone of brain natriuretic peptide (NT-proBNP) and highly sensitive (hs) troponin I, resting 12-leads electrocardiography (ECG), echocardiography (ECHO)) parameters were regularly analysed.

Eligible patients had a confirmed diagnosis of FD by low enzymatic activity (males) and by identifying a disease-causing mutation (males and females). The enzymatic activity was measured in leucocytes or in dried blood spots (DBS). Pathogenic mutation was recognized by *GLA* gene sequencing. The assessment was performed in the different laboratories (in Slovakia or abroad).

The study was approved by the Ethics Committee of National Institute of Children’s Diseases in Bratislava (R868/2018/OPPaM-HK), and was conducted in accordance with institutional guidelines. Written informed consent was obtained from all patients included in this study.

Descriptive statistical analyses were performed with Microsoft Excel (version 2203, Microsoft Corporation, Redmond, WA, USA).

## 3. Results

To provide optimal care for and management of FD patients in Slovakia, they were centralised in one place—specifically, in the Centre for Inborn Errors of Metabolism in National Institute of Children’s Diseases in Bratislava.

Since 1999, when the first FD patient was diagnosed, until 2021, 48 FD patients have been collected in our centre. Among them, there are 21 males and 27 females. Nine patients (five girls, four boys) were recognised in childhood by the pedigree analysis. Up to now, three males and one female have died. In our cohort, we have identified five patients with as-yet undescribed mutations: one family (mother, son, granddaughter) with frameshift mutation c.736del with a classic phenotype, one woman with missense mutation c.157A>C, and one woman with missense mutation c.203T>C.

**Figure 1:**
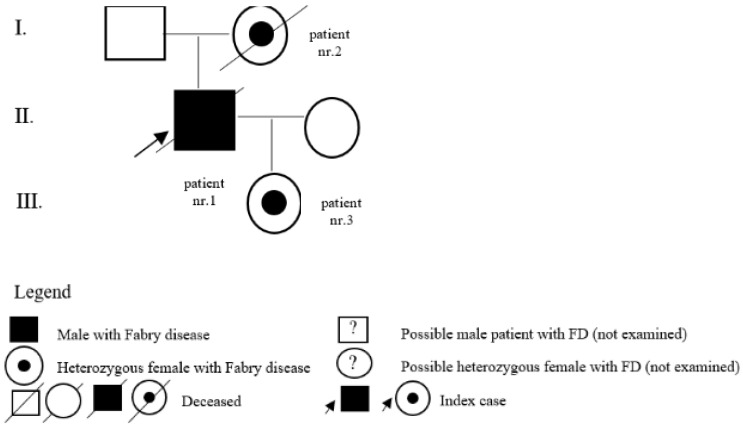
Pedigree of family with mutation c.128G>A.

**Figure 2:**
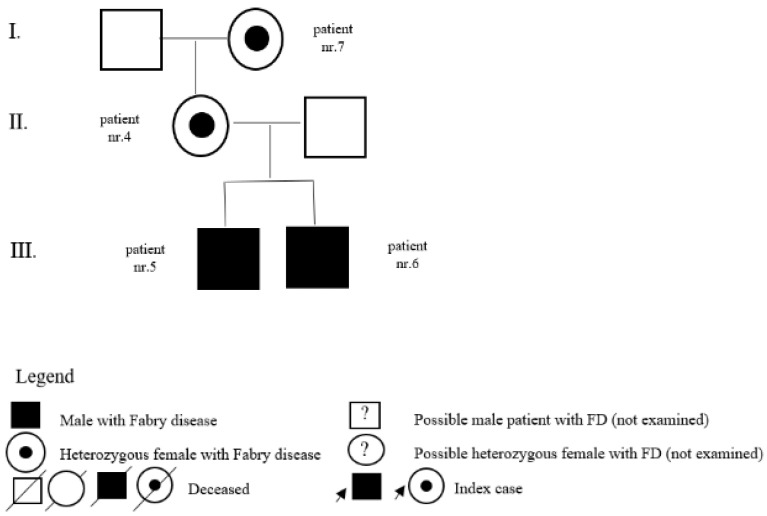
Pedigree of family with mutation c.463G>C.

**Figure 3:**
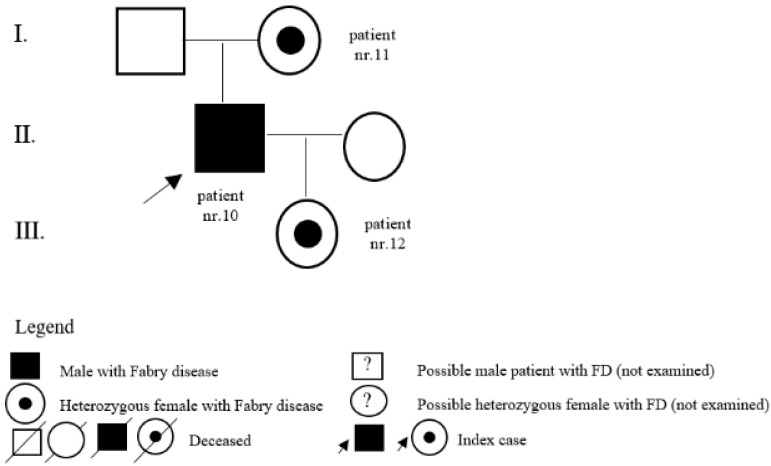
Pedigree of family with undescribed mutation c.736delA.

**Figure 4:**
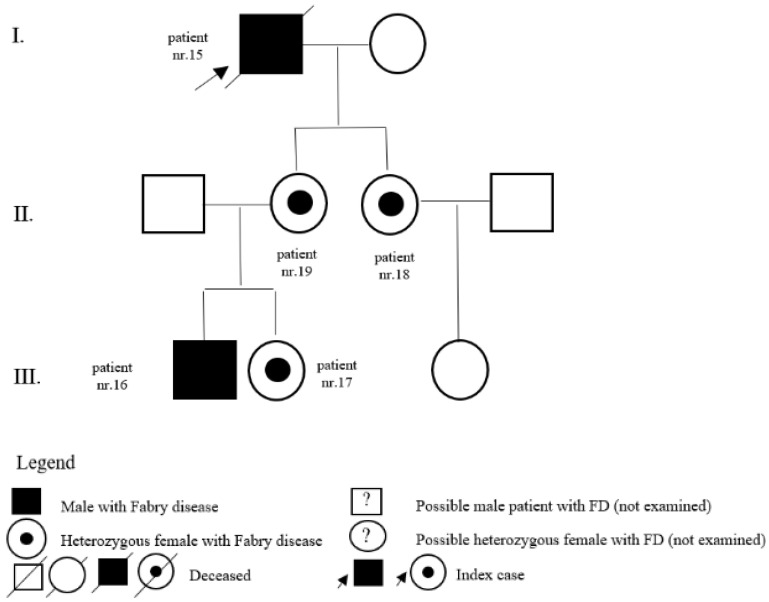
Pedigree of family with mutation c.98A>G.

**Figure 5:**
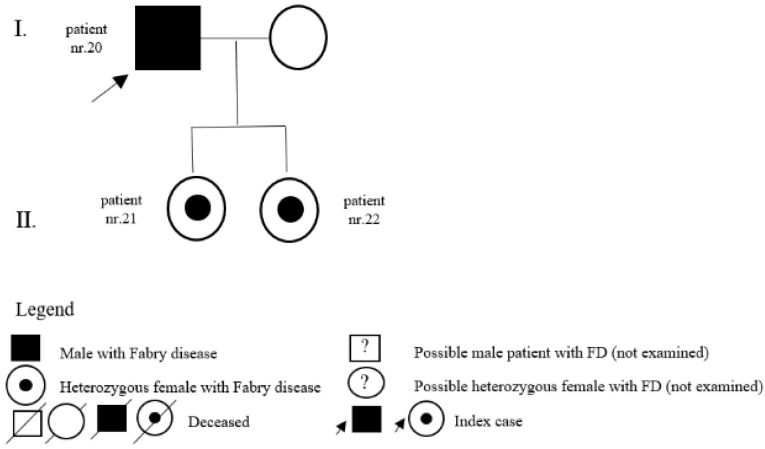
Pedigree of family with mutation c.334C>T.

**Figure 6:**
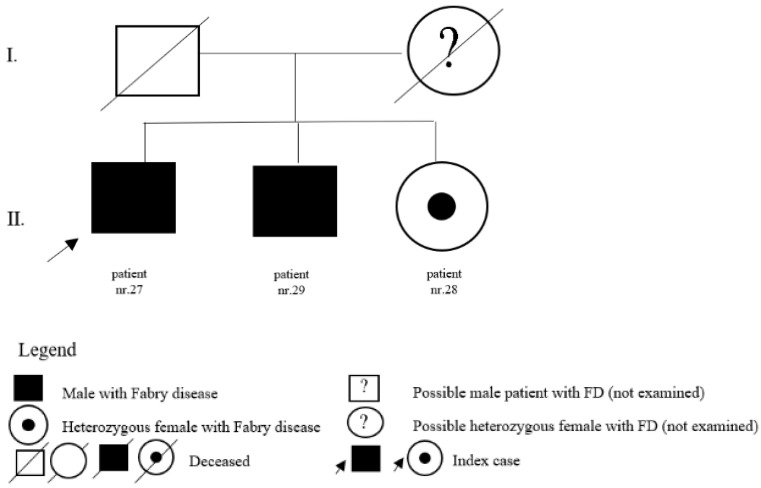
Pedigree of family with mutation c.758T>C.

**Figure 7:**
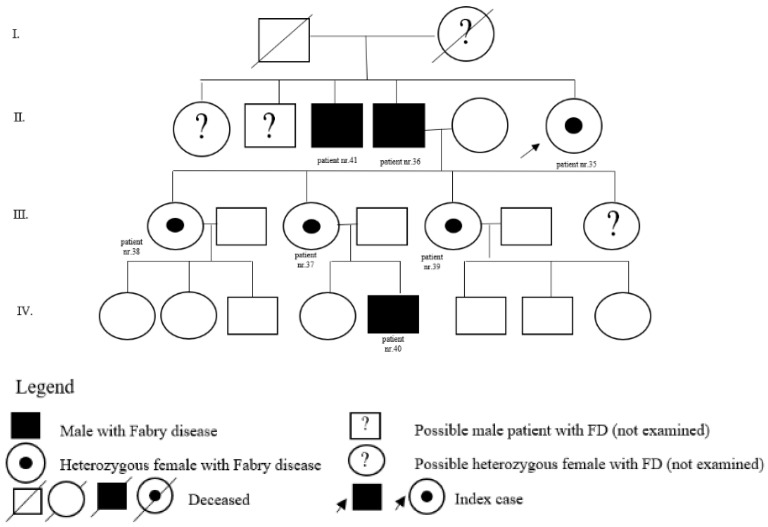
Pedigree of family with GVUS c.427G>A (p.Ala143Thr).

**Figure 8:**
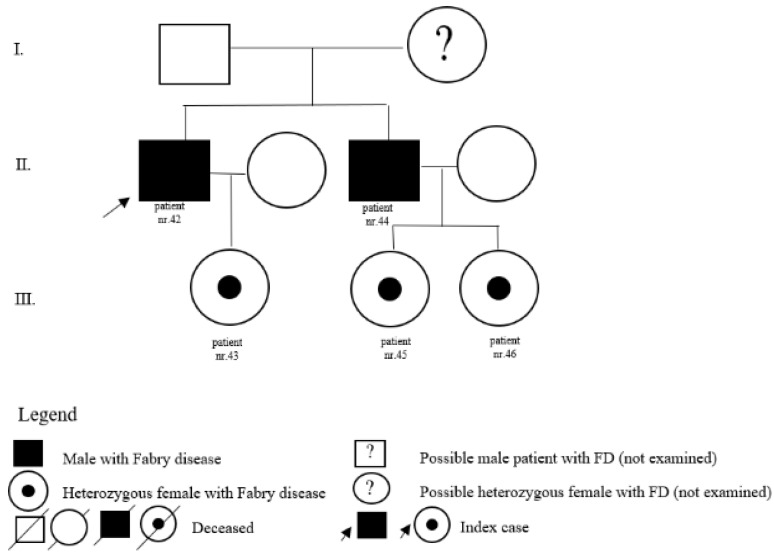
Pedigree of family with GVUS c.427G>A (p.Ala143Thr).

In the register of our centre, there are also patients with a variant of unclear significance or a probably benign variant: 14 (5 M, 9 F) with p.A143T and 3 (2 M, 1 F) with p.R118C. Patients diagnosed with p.D313Y are actually considered as not pathogenic, or as a polymorphism variant, and they are followed by their local physician. Complete results with *GLA* variant are shown in Table 1. Pedigrees of families with different mutations and with GVUS are demonstrated in the following Figure 1, Figure 2, Figure 3, Figure 4, Figure 5, Figure 6, Figure 7 and Figure 8.

In 2021, Slovakia had a population of 5,449,270 inhabitants [19]; the prevalence of the disease in our country can be estimated at 1:113,526.

The first FD patient in Slovakia was diagnosed in 1999. In a 20-year-old male with proteinuria, a kidney biopsy revealed a typical histological image of FD. The diagnosis was confirmed with an examination of the enzyme activity of α-Gal A and genetic testing.

His mother was the second Slovak FD patient. She suffered from LVH and cardiological impairment. Following the years 2000–2007, only a few patients (seven) were diagnosed. Among them, two boys, their mother, and their grandmother were diagnosed with FD through a family study during a nationwide screening programme organised in the Czech Republic.

Since 2008, the examination of the enzymatic activity of α-Gal A in DBS is available in our country. This opened an opportunity to commence the Slovak screening programmes in high-risk populations. These screening programmes are organised with collaborations between cardiologists, nephrologists, and neurologists.

Primary activities are focused on common clinical symptoms connected with FD. Patients with LVH, patients with renal impairment (after renal transplant, predialysed, dialysed), young patients with stroke, and patients with sclerosis multiplex are of great concern.

This approach has led to the identification of 16 index cases with a *GLA* gene variant. Pedigree analysis and subsequent cascade family genetic testing were performed, leading to the identification of another 23 relatives carrying a pathogenic gene variant.

An analysis of the leading clinical signs revealed that nine (six males, three females) patients were recognised by the study in patients with LVH. Five (two males, three females) cases were among the patients with renal impairment, and two (zero males, two females) cases were found in the population with sclerosis multiplex.

Since 2003, when the first patient started the treatment with ERT, 14 patients (11 males and 3 females) have been treated overall, all of them with ERT. Our experience with ERT is associated with important benefits that preserve critical organ function, including the stabilization of renal function and the prevention of end-stage renal disease, an improvement in cardiac structure and function, a reduction in neuropathic pain severity, and an improvement in gastrointestinal symptoms. We have observed an at least temporary improvement of the clinical status in all of the Slovak treated cohort. However, we must admit that the ERT has its limits.

Regretfully, three treated patients died. One 69-year-old woman died due to heart failure after seven years of therapy. She achieved temporary improvement in the self-well-being (SWB) and cardiac parameters. Due to the fact that the treatment was started in the terrain of LVH, the progression was irreversible. Her son was the first Slovak FD patient; he died at the age of 37 after an unexpected, ruptured brain aneurysm. He started ERT as a 24-year-old and was the only FD patient in our cohort that underwent a kidney transplant at the age of 30. Apparently, the treatment came too late to stop the organ failure progression. After the renal transplantation, his renal parameters remained stable, thanks to the continuation of the treatment. Another man died at 62 years old, after eight years of therapy, due to multiorgan failure after repeated stroke. This case describes the limitation of ERT. Agalsidase treatment does not cross the blood–brain barrier. Despite the fact that there are data supporting the beneficial effect of ERT for stroke prevention [20], some patients may present with repeated cerebrovascular events.

Among the patients recognised in childhood, three boys have started treatment at the ages of 18, 14, and 13 years, respectively. The median (range) age at symptom onset, age at diagnosis, and age at treatment in treated patients is 14.0 (10.0–54.0); 36.5 (4.0–54.0), and 38.0 (13.0–65.0) years, respectively. The average (range) time from the first symptom to diagnosis is 14.8 (−8.0–45.0) years, and the average (range) time from diagnosis to treatment is 3.0 (0.0–14.0) years. Among the patients recognised in childhood, three boys started treatment at ages of 18, 14, 13 years, respectively. The average (range) time of duration of ERT is 6.0 (1.0–16.0) years. The type of the treatment and the effect of the treatment are shown in Table 2.

Patients who are indicated for the treatment and are not receiving it either refused it or are waiting for confirmation by the regulatory body.

## 4. Discussion

This study is the first complex report mapping the situation surrounding FD patients in Slovakia.

FD belongs to the most-frequent LSDs (lysosomal storage disorders). Moreover, it is reported as the most-frequent LSD among adult patients [4]. In consideration of more treatment options and with more emphasis being placed on personalized medicine in recent years, we have realised the necessity for such analysis.

During the period of 23 years (1999–2021), we collected 48 patients with FD in the Centre for Inborn Errors of Metabolism in National Institute of Child Diseases in Bratislava. The estimated prevalence of FD in Slovakia is 1:113,526. In the Czech Republic (our neighbouring country, with whom we formed Czechoslovakia in the past, with approximately twice the population of Slovakia), the estimated prevalence of FD was 1:76,000, as reported in 2017 [21]. On the other hand, in Poland (our neighbouring country to the north, with a population of 38.2 million) the prevalence of FD, as reported in Polish studies, is 2.5/100,000 in the entire population: 1/40,000 in men and 0.85/100,000 (1/117,000) in women. [22]

The historical prevalence of FD was previously estimated to 1:117,000 [15], whereas recent neonatal screening programs demonstrated a much higher incidence [16,23,24]. Although neonatal screening programs are feasible, they result in the identification of many variants of unknown significance or benign variants. Therefore, many studies were trying to establish the prevalence of FD in populations with organ manifestations typical for FD [1,3,25,26,27]. These included patients (the so-called high-risk population) with end-stage renal disease, early stroke, unexplained left ventricular hypertrophy and/or hypertrophic cardiomyopathy, and sclerosis multiplex.

Live births registered at the Statistical Office of the Slovak Republic in the period from 1999 to 2021 totalled 1,230,430 [19,28]. The total prevalence of FD in the Slovak population can be estimated at 3.9:100,000 live births. Excluding GVUS (p.R118C and p.A143T, overall, 17 patients), the prevalence decreases to 2.5:100,000 live births.

The prevalence increased after the start of the screening studies in the high-risk population in 2008, from 1.66 to 5.34 or 3.07 (excluding GVUS) per 100,000 live births.

In our neighbouring countries of Austria and Hungary, pilot studies for newborn screening for lysosomal storage disorders took place, and the birth incidence for FD was 1:3859 (including p.A143T) [29] and 1:13,341 (including p.A143T present in all three diagnosed patients) [30], respectively. In one of the first newborn studies, published by Spada et al., the incidence of FD was reported as 1:3100 [23], and included newborns diagnosed with *GLA* variants which are currently considered as not disease-causing (p.E66G, p.A143T, p.R118C). The exclusion of these patients reduces the incidence to an estimate of 1:5000 [31]. These findings supported other studies conducted in different countries [18,23]. Newborn screening for FD has not been conducted in Slovakia.

Generally, we can consider that the disease is underdiagnosed in our country. Moreover, our observation, searching in family history, has shown that lot of diagnosed Slovak FD patients had parents, aunts, uncles, or other family members that died prematurely due to stroke or heart or kidney failure. However, the lack of awareness about the disease and the complicated approach to diagnostic methods in the past have caused misdiagnoses and failures to recognize the disease entirely.

So far, we have recorded 31 patients with a pathogenic mutation. A total of 26 patients have a pathogenic variant already described. Five patients remain with as-et undescribed mutations. One family (mother, son, granddaughter) with frameshift mutation c.736del (p.Thr246HisfsTer23) is classified as a pathologic variant with the classic phenotype. The son, a 25-year-old man, has been diagnosed with classic presentation of FD since childhood (acroparesthesia, angiokeratoma, cornea verticillata, proteinuria). He has had a positive bioptic finding with typical zebra bodies in kidney biopsy. The low enzyme activity of α-GAL A and genetic analysis confirmed the diagnosis. LysoGB3 as biomarker remains slightly elevated despite ERT treatment. His clinical status at this time is stabilised. His mother began to have cardiological problems at the age of 59. She has normal enzyme activity of α-GAL A and higher level of LysoGB3. She is not treated yet. His daughter, at the age of one year, is asymptomatic.

One woman with missense mutation c.157A>C (p.Asn53His) was diagnosed in a screening conducted among patients with sclerosis multiplex. She was diagnosed with multiple sclerosis that did not respond to treatment. Arrhythmia has been added to the neurological problems. Acral pain during childhood was described. A 42-year-old woman was diagnosed with FD, and she started ERT treatment. She has LysoGB3 in the reference range. This variant is classified as likely pathogenic, and in the same position, pathogenic variants p.Asn53Asp [32] and p.Asn53Lys [33] were described.

One 46-year-old woman with missense mutation c.203T>C (p.Leu68Pro) was diagnosed in a screening with hypertrophic cardiomyopathy. She has a high level of LysoGB3 and cardiac impairment. She is not treated yet. This variant is classified as likely pathogenic, and in the same position, pathogenic variant p.Leu68Phe was described previously [34].

To the best of our knowledge, there is scarce information about genetic variants in the *GLA* gene c.801+1G>A (p.L268IfsTer3) found in one of our patients. This splicing defect is classified as pathogenic in the VarSome database, and as likely pathogenic in the ClinVar database. Li et al. described a Chinese patient with renal impairment [35].

Another patient from our cohort was diagnosed with FD as 55-year-old man suffering from acral pain since childhood, worsening with warmth. He suffers from hypoacusis as well. At the age of 49 years, hypertension and hypertrophic cardiomyopathy appeared. Low enzyme activity of α-GAL A and a high level of LysoGB3 was found. He has been treated for 3.5 years with ERT, but now we observe signs of cardiac failure in him. On the other hand, his renal parameters are in the reference range.

### 4.1. Fabry Disease in Females

For many years, heterozygous females with Fabry disease were thought to be the only carriers of the disease. More recently, increasing evidence has emerged that those females with Fabry disease manifest clinical symptoms and have a reduced life expectancy compared to healthy subjects; but they differ from classic male patients. Firstly, females with Fabry disease have a wider spectrum of disease severity, ranging from asymptomatic to severely affected phenotype (rare). The pattern and severity of the organ involvement depends on the *GLA* mutation and the X-chromosome inactivation pattern.

Therefore, females are mosaic and consist of a population of cells with preferential expression of either a paternal or maternal X-chromosome. Not all females have equal proportions of cells with the paternal or maternal X-chromosome inactivated. This is the so-called skewed X-inactivation [36,37,38].

Secondly, females sometimes present with symptoms in a single organ, rather than the multisystemic manifestations observed in classic male cases. Furthermore, contrary to males, females may develop myocardial fibrosis before LVH, suggesting that LVH may not be the only driver of the cardiac disease burden in females [9].

In our FD group, we record 27 female patients: 17 with a pathologic variant, 10 with GVUS. Three patients were diagnosed in screening studies with hypertrophic cardiomyopathy, and one in a screening with sclerosis multiplex. Thirteen patients were diagnosed in a family screening. One of them was symptomatic with cardiac impairment. Four patients with a pathogenic variant have been treated with ERT so far, three of them due to cardiological impairment, and one with neurological and cardiological impairment. One treated woman died.

### 4.2. Genetic Variants of Unknown Significance (GVUS)

There is ongoing debate about the pathogenicity and phenotypic manifestation of several GVUS, including variants found in our cohort: p.R118C, p.A143T.

The p.A143T variant was firstly described in 1997, and its phenotypic expression was concluded by the authors as being unknown [39]. Later, the variant was classified as a later-onset pathogenic mutation [40,41]. Based on the clinical information, the variant has been reclassified [42,43], and although it is still a matter of debate, p.A143T seems to be most likely a neutral variant or a possible modifier, instead of a disease-causing mutation [43]. It is currently referred to as GVUS or a probably benign variant [44].

The p.R118C variant was primarily identified in a neonatal screening in 2004 [45]. Based on the biochemical characteristics of the enzyme, the later-onset FD phenotype was predicted [23]. Later, the variant was reclassified to GVUS [42,45].

In the register of our centre are 14 patients (5M,9F) with p.A143T, and 3 (2M,1F) with p.R118C. None of these patients are receiving ERT. From studies in high-risk populations, we observed an increasing prevalence of FD, but also rising numbers of GVUS.

Interestingly, in our cohort of patients, GVUS are identified mainly among patients with renal impairment (five) and only in one patient with sclerosis multiplex. There have been none identified in the LVH group.

Recently, published data from the Nationwide Neurological Screening of Stroke Patients in the Czech Republic identified 23 individuals (16 index patients and 7 positively tested family members) carrying 8 different *GLA* gene variants. Pathogenic mutations were detected in 2 index patients and their 3 relatives, and GVUS or likely benign variants in 14 index patients and their 4 relatives [25]. Interestingly, in our cohort, there are no patients discovered in the study with stroke.

Various studies in multiple countries have reported the prevalence of newly identified Fabry disease to be approximately 0–1.17% in chronic kidney disease patients. In our record, there are only two patients diagnosed with FD, primarily due to renal impairment. These patients were found before the screening in high-risk population had started. However, 3 patients (1 M, 2 F) with the p.A143T genotype, 2 patients (1 M, 1 F) with the p.R118C genotype, their relatives (10 patients; 4 M, 6 F) with p.A143T genotype, and 1 male patient with the p.R118C genotype were identified during different studies organised in Slovakia among patients with renal impairment. In one family with the p.A143T genotype including nine members (six females, three males), we found more detailed information. A 73-year-old female was discovered in a study performed among dialysed patients. Other data were not available. All (three) identified men in the pedigree analysis have/had low enzyme activity of α-GAL A. One of them, a 14-year-old boy, has undergone a series of examinations, with the result of juxtacortical frontal white matter lesions bilaterally. He experienced the depressed mood often reported in FD patients, which was resolved after psychological therapy. His grandfather has been suffering from hypertension since the age of 70; otherwise, he was in good condition. He was 78 years old at the time of the diagnosis of FD and refused further examinations. A mother of a 14-year-old boy and her two sisters underwent cardiological, nephrological, and neurological examinations without any clinical signs of FD.

### 4.3. Treatment of Fabry Disease

The current specific therapy for FD includes intravenously administered ERT with recombinant agalsidase alpha or beta (every other week), or oral pharmacological chaperone therapy with migalastat (every other day) in patients with amenable mutations [46]. Several treatments, including modified enzymes, substrate reduction therapy, and gene therapy, are in development [47,48,49]. Many studies with long-term data have shown the positive effect of ERT [50,51,52]. The efficacy of migalastat has been proven and published as well [53,54]. Overall, 14 patients (11 males and 3 females) have been treated in Slovakia, all of them with ERT.

Current data recommend the initiation of treatment in any male patients with classical FD aged at least 16 years of age, and in younger males with classical disease if early signs of organ damage appear [55]. Consensus recommendations for the treatment of FD in paediatric patients published in 2019 propose that ERT initiation should be considered for symptomatic boys and girls, and that prophylactic ERT treatment be considered for asymptomatic boys from the age of seven [31]. The first treated boy in our cohort was diagnosed at the age of four through family screening, and started treatment as an 18-year-old man, due to the deterioration of the renal parameter (albuminuria) and psychiatric problems (depression). In that time, LysoGB3 examination or T1 mapping in cMRI (cardiac magnetic resonance imaging) were not ordinarily available. His younger brother started treatment sooner, at the age of 13, due to a rising level of LysoGB3 and mood problems. Our last treated boy started therapy at the age of 14 for the same reasons as the previous one. The initiation of therapy in asymptomatic children is not an easy decision. This will not only fundamentally affect the individual’s childhood, but also the functioning of the whole family. Until recently, there was only one approved treatment, which required the child to have an infusion of the enzyme every other week. Actually, the administration of migalastat with a Peroral chaperon is available for patients with amenable mutations aged 16 years old in the U.S. [56] and 12 years old in Europe [57]. In Slovakia, we do not yet have experience with the use of Migalastat. Indeed, newly published data supports and encourages us to start the treatment earlier.

A recent study has shown the importance of the timing of the treatment initiation. It is crucial to achieve favourable benefits from therapy [58]. The ongoing heated debate among worldwide FD specialists is concerned with how early is early enough to prevent irreversible organ damage. Various recommendations were published [13,31,55,59,60]. Early indicators of disease progression in FD that may indicate the need for disease-specific treatment initiation were published by panel of clinician-experts in FD in 2020. The 27 indicators include early renal, cardiac, peripheral nervous system, and patient-reported signs and symptoms [10]. The reduced myocardial T1 relaxation time in cardiac MRI, which is able to detect the prehypertrophic stage of accumulation in the heart [61,62,63], has come to the fore [10,14]. This very promising examination is also available in Slovakia. We expect it will help us to monitor all FD patients, particularly children (mainly boys) and women who are waiting for correct therapy initiation and for better follow-ups with treated men. Increased attention is given to gastrointestinal symptoms and to neuropathic pain, representing early signs of FD [10,64].

Starting with ERT treatment in 2003 in Slovakia, at the beginning, the first two patients were admitted into the hospital due to possible adverse reactions to infusion. Further patients have started treatment in the centre, and after 1–2 months, we organised an application site (hospital or designated outpatient settings) near the patients’ homes. In contrast with other countries, home infusions for FD treatment are not available in Slovakia [65]. To avoid adverse event reactions during the first 6–12 months, we use premedication (antipyretics, antihistaminic). Over 18 years of treatment, only a few adverse reactions (such as rash, fever) have been observed. During the course of ERT, anti-agalsidase antibodies may be developed, which can affect the outcome of the therapy [13]. The routine evaluation of these antibodies is not available in Slovakia. Agalsidase alfa is given at 0.2 mg/kg body weight every other week by intravenous (IV) infusion and agalsidase beta is administered at 1.0 mg/kg body weight once every 2 weeks as an IV infusion. In Slovakia, in case of an inadequate response to ERT, we are not allowed to modify the treatment regime.

Early diagnosis and the right timing of therapy are the key factors for the modification of the natural course of FD. Even though FD is a chronic, progressive disease, it generally manifests without cognitive or physical impairment. Patients suffering from FD are often educated and are part of the labour market, where they can, despite their disease, create economical and mental value.

In 2016, during our local Czechoslovak conference engaged in inborn errors of metabolism, we reported our results from a quality-of-life assessment using a WHOQOL-BREF questionnaire among adult patients with lysosomal storage disorders. All FD adult patients (seven) included in the study have completed high school or have a university education, and they were all employed (except for one). Moreover, they reported the highest quality of life in comparison with other adult patients with LSD Gaucher disease type 1 (nine patients); Pompe disease (four patients); Mucopolysaccharidosis (three patients); and Niemann–Pick type C (seven patients) [66].

We consider this centralized system of FD care to be effective, with highly satisfactory access to correct diagnostics, follow-ups, and therapy. Some smaller countries in Europe, including Slovenia, the Czech Republic, Lithuania, and Hungary, have centralized FD patient care systems, with a single specialized national centre in each country [67].

Our centre tries to offer not only complex multidisciplinary care for FD patients but also educational activities for medical professionals.

Thanks to the developing therapeutical options (ERT, chaperons, next-generation ERTs, substrate reduction therapy, gene therapy), the treatment of patients with FD is tailored according to their specific needs.

In addition to causal FD treatment, the need for the concomitant medication of affected patients is strongly indicated. Management of the renal, cardiac, neurological, gastroenterological, and other complications with pharmacological and non-pharmacological treatment methods goes hand-in-hand with specific therapies for Fabry disease [13,68].

## 5. Conclusions

Increasing knowledge about inborn metabolic errors has changed the spectrum of the patients. However, early diagnostics improves treatment and brings greater survival to FD patients diagnosed in childhood. In our study, we have confirmed that active screenings in the high-risk populations have resulted in an increasing number of adult patients with FD in recent years. This fact creates a new challenge for adult physicians. Moreover, we have shown that, in the Slovak population, a kinship analysis works effectively in discovering new patients.

According to the literature, late-onset forms of the disease are more frequent than the classic phenotype. We did not confirm these results in our group of patients. It is true that the majority of cases were diagnosed in an adult age, but the reason was diagnostic delay (most of them have a pathogenic *GLA* variant classified as a classic phenotype with the clinical signs of FD since childhood).

Considering the complexity of the FD topic, it is of great importance to share patient data. In order to better understand GVUS, all variant carriers should be followed and evaluated prospectively. These data, in the future, will help to distinguish symptoms attributable to FD from nonspecific comorbidities in benign *GLA* variant carriers.

This study depicts the overview of FD in Slovakia. It brings information about three new pathogenic variants that have not been published before. Moreover, it describes the new point of view of the variant (c.801+1G>A) previously linked to the renal phenotype only.

Following the example of the other European countries, in the future, we would like to provide the possibility of home-based infusion treatment to our patients, which has been shown to be a safe and convenient alternative to clinic-based treatments. This may also serve as a cornerstone for home-based treatment programs in other rare diseases, and it can help to avoid treatment disruptions in some unexpected scenarios, such as what we had during the COVID-19 pandemic. In the near future, we are planning to commence chaperone therapy, and we hope that novel therapies, including gene therapy, will be also available in Slovakia.

Our study confirms the effectiveness of ERT. We have observed at least temporary improvement in all our patients, mainly regarding the amelioration of SWB, while taking into account its limitations. In our centre, we are now focusing on early signs of organ impairment in order to achieve the right timing of starting the therapy. Our effort is aimed at children and young adults, with particular concern for female patients, who will profit the most from early treatment, before the occurrence of irreversible organ damage.

## Figures and Tables

**Table 1:** Data of 48 Slovak FD patients.

Nr.	*GLA* Variant	Sex	Age at Diagnosis (Years)	Phenotype Previously Described	Type of Screening	Therapy	Age at Treatment (Years)	Comment
Patient with pathogenic variant								
1	c.128G>A (p.Gly43Asp)	M	20	classic	kidney	Y	24	son of patient nr. 2 and father of patient nr. 3
2	c.128G>A (p.Gly43Asp)	F	50	classic	pedigree	Y	62	mother of patient nr. 1
3	c.128G>A (p.Gly43Asp)	F	0	classic	pedigree	N		daughter of patient nr. 1
4	c.463G>C (p.D155H)	F	23	classic	pedigree	N		mother of patients nr. 5 and nr. 6, daughter of patient nr. 7
5	c.463G>C (p.D155H)	M	4	classic	pedigree	Y	18	brother of patient nr. 6
6	c.463G>C (p.D155H)	M	5	classic	pedigree	Y	13	brother of patient nr. 5
7	c.463G>C (p.D155H)	F	52	classic	pedigree	N		mother of patient nr. 4
8	NA	M	29	NA	NA			
9	NA	M	35	NA	NA	Y	35	
10	c.736delA (p.Thr246HisfsTer23)	M	25	undescribed/classic	kidney	Y	25	son of patient nr. 11 and father of patient nr. 12
11	c.736delA (p.Thr246HisfsTer23)	F	59	undescribed/classic	pedigree	N		mother of patient nr. 10
12	c.736delA (p.Thr246HisfsTer23)	F	0	undescribed/classic	pedigree	N		daughter of patient nr. 10
13	c.169C>T (p.Q57X)	M	54	classic	LVH	Y	54	father of patient nr. 14
14	c.169C>T (p.Q57X)	F	24	classic	pedigree	N		daughter of patient nr. 13
15	c.98A>G (p.D33G)	M	58	classic	LVH	N		father of patients nr. 18 and nr. 19, grandfather of patients nr. 16 and nr. 17
16	c.98A>G (p.D33G)	M	8	classic	pedigree	Y	14	son of patient nr. 19 and brother of patient nr. 17
17	c.98A>G (p.D33G)	F	15	classic	pedigree	N		daughter of patient nr. 19 and sister of patient nr. 16
18	c.98A>G (p.D33G)	F	31	classic	pedigree	N		daughter of patient nr. 15
19	c.98A>G (p.D33G)	F	33	classic	pedigree	N		daughter of patient nr. 15, mother of patients nr. 16 and nr. 17
20	c.334C>T (p.R112C)	M	35	classic	LVH	Y	35	father of patients nr. 21 and nr. 22
21	c.334C>T (p.R112C)	F	0	classic	pedigree	N		daughter of patient nr. 20
22	c.334C>T (p.R112C)	F	0	classic	pedigree	N		daughter of patient nr. 20
23	c.334C>T (p.R112C)	M	38	classic	LVH	Y	38	
24	c.730G>A (p.Asp244Asn)	F	50	classic	LVH	Y	51	
25	c.1073A>G (p.Glu358Gly)	F	85	classic	LVH	N		
26	c.801+1G>A, (p.L268IfsX3)	M	55	renal	LVH	Y	56	new description of phenotype—arrythmia, LVH, depression
27	c.758T>C (p.Ile753Thr)	M	65	classic	LVH	Y	65	brother of patients nr. 28 and nr. 29
28	c.758T>C (p.Ile753Thr)	F	55	classic	pedigree	N		sister of patient nr. 27 and nr. 29
29	c.758T>C (p.Ile753Thr)	M	62	classic	pedigree	N		brother of patients nr. 27 and nr. 28
30	c.157 A>C (p.Asn53His)	F	42	undescribed	SM	Y	43	arrythmia, neurological impairment
31	c.203T>C (p.Leu68Pro)	F	46	undescribed	LVH	N		hypertrophic cardiomyopathy
Data of 48 Slovak FD patients—part 2								
Patients with GVUS								
32	c.352C>T (p.R118C also p.Arg118Cys)	F	75		kidney	N		mother of patient nr.33
33	c.352C>T (p.R118C also p.Arg118Cys)	M	45		pedigree	N		son of patient nr. 32
34	c.352C>T (p.R118C also p.Arg118Cys)	M	41		kidney	N		
35	c.427G>A(p.A143T also p.Ala143Thr)	F	73		kidney	N		sister of patient nr. 36
36	c.427G>A(p.A143T also p.Ala143Thr)	M	78		pedigree	N		brother of patient nr. 35
37	c.427G>A(p.A143T also p.Ala143Thr)	F	43		pedigree	N		daughter of patient nr. 36
38	c.427G>A(p.A143T also p.Ala143Thr)	F	46		pedigree	N		daughter of patient nr. 36
39	c.427G>A(p.A143T also p.Ala143Thr)	F	49		pedigree	N		daughter of patient nr. 36
40	c.427G>A(p.A143T also p.Ala143Thr)	M	14		pedigree	N		son of patient nr. 37
41	c.427G>A(p.A143T also p.Ala143Thr)	M	62		pedigree	N		brother of patient nr. 35
42	c.427G>A(p.A143T also p.Ala143Thr)	M	73		kidney	N		father of patient nr. 43 and brother of patient nr. 44
43	c.427G>A(p.A143T also p.Ala143Thr)	F	43		pedigree	N		daughter of patient nr. 42
44	c.427G>A(p.A143T also p.Ala143Thr)	M	69		pedigree	N		father of patients nr.45 and nr. 46 and brother of patient nr. 42
45	c.427G>A(p.A143T also p.Ala143Thr)	F	45		pedigree	N		daughter of patient nr. 44
46	c.427G>A(p.A143T also p.Ala143Thr)	F	46		pedigree	N		daughter of patient nr. 44
47	c.427G>A(p.A143T also p.Ala143Thr)	F	82		kidney	N		
48	c.427G>A(p.A143T also p.Ala143Thr)	F	31		SM	N		

Legend: Nr. = patient number; F = female; M = male; LVH = patient identified in screening study with left ventricular hypertrophy; kidney = patient identified in screening study with kidney impairment; SM = patient identified in screening study with sclerosis multiplex; pedigree = patient identified during family screening; Y = yes; N = No.

**Table 2:** The characteristics of the treated patients.

Nr.	Variant	Sex	Age at First Symptom (Years)	Age at Diagnosis (Years)	Phenotype Previously Described	Age at Treatment (Years)	Time from First Symptom to Diagnosis	Time from Diagnosis to Treatment	Therapy	Duration of ERT (Years)	Effect of Therapy	LysoGb3 Prior ERT	LysoGb3 after ERT (Last Value)	Age at Death (Years) and Reason
1	c.128G>A (p.Gly43Asp)	M	14	20	classic	24	6	4	ERT (agalsidase beta, agalsidase alfa)	13	stabilization of renal parameters; SWB improved	NA	NA	37; ruptured brain aneurysm
2	c.128G>A (p.Gly43Asp)	F	54	50	classic	62	−4	12	ERT (agalsidase alfa)	7	temporary improvement of CV parameters, improvement of physical activities	NA	6.0	69; heart failure
3	c.463G>C (p.D155H)	M	12	4	classic	18	−8	14	ERT (agalsidase beta)	8	stabilization and improvement of renal and CV parameters, SWB improved	NA	7.9 (N 0–3.5) ng/mL	
4	c.463G>C (p.D155H)	M	13	5	classic	13	−7	8	ERT (agalsidase beta)	4	renal and CV parameters in reference level; normal psychomotor development	142.9 (N 0–3.5) ng/ml	11.1 (N 0–3.5) ng/mL	
5	NA	M	15	35	NA	35	20	0	ERT (agalsidase beta)	NA	NA	NA	NA	
6	c.736delA (p.Thr246HisfsTer23)	M	14	25	undescribed/classic	25	11	0	ERT (agalsidase beta)	16	stabilization and improvement of renal parameters; pain relief; SWB improved	NA	25.1 (N 0–3.5) ng/mL	
7	c.169C>T (p.Q57X)	M	35	54	classic	54	19	0	ERT (agalsidase alfa)	8	temporary improvement of CV parameters, stabilization of renal parameters, SWB improved	NA	40 (N 0–1.8) ng/mL	62; multiorgan failure after repeated stroke
8	c.98A>G (p.D33G)	M	14	8	classic	14	−6	6	ERT (agalsidase alfa)	2	stabilization of renal parameters; normal psychomotor development	38 (N 0–1.8) ng/ml	11.7 (N 0–1.8) ng/mL	
9	c.334C>T (p.R112C)	M	14	35	classic	35	21	0	ERT (agalsidase alfa)	9	stabilization and improvement of renal and CV parameters; SWB improved	NA	14.7 (N 0–3.5) ng/mL	
10	c.334C>T (p.R112C)	M	10	38	classic	38	28	0	ERT (agalsidase beta)	3	stabilization of renal and CV parameters, improvement of physical activities	89.5 (N 0–3.5) ng/ml	16.0 (N 0–3.5) ng/mL	
11	c.730G>A (p.Asp244Asn)	F	16	50	classic	51	34	1	ERT (agalsidase alfa)	1	stabilization and improvement of renal and CV parameters; SWB improved	5.7 (N 0–1.8) ng/mL	4.2 (N 0–1.8) ng/mL	
12	c.801+1G>A, (p.L268IfsX3)	M	10	55	renal	56	45	1	ERT (agalsidase alfa)	3.5	stabilization of renal parameters; end of ERT after 3.5 years, due to deterioration of cardiological findings	95.8 (N 0–1.8) ng/mL	48.1 (N 0–1.8) ng/mL	
13	c.758T>C (p.Ile753Thr)	M	64	65	classic	65	1	0	ERT (agalsidase beta)	3	stabilization of renal and improvement of CV parameters, improvement of physical activities	18.1 (N 0–3.5) ng/mL	8.6 (N 0–3.5) ng/mL	
14	c.157 A>C p.(Asn53His)	F	16	42	undescribed	43	26	1	ERT (agalsidase alfa)	1	stabilization of renal and CV parameters; improvement in gross motor function and self-activity	1.3 (N 0–1.8) ng/mL	1.0 (N 0–1.8) ng/mL	

Legend: M = male; F = female; ERT = enzyme replacement therapy; SWB = subjective well-being; CV = cardiovascular; NA = not available; CV parameters = N-terminal prohormone of brain natriuretic peptide (NT-proBNP) and highly sensitive (hs) troponin I, resting 12-leads electrocardiography (ECG), echocardiography; renal parameters = serum creatinine, total protein in urine and urine albumin-to-creatinine ratio, estimated glomerular filtration rate (eGFR, mL/min/1.73 m^2^).

## Data Availability

The data that supports the findings of this study are available from the corresponding author on reasonable request.
